# Supplementary material for: neuroWalknet, a controller for hexapod walking allowing for context dependent behavior
Source: PLoS Comput Biol. 2023 Jan 24;19(1):e1010136. doi: 10.1371/journal.pcbi.1010136 (PMC9897571; doi:10.1371/journal.pcbi.1010136)
Supplement: S1 File — (PDF) [file pcbi.1010136.s001.pdf]

Supporting Information

S1 PDF – **Additional Experimental Data for Standing and Searching Movements**

in Malte Schilling and Holk Cruse (2022): neuroWalknet, a controller for hexapod walking allowing for context dependent behavior. *PLOS Computational Biology*

As supporting information, we provide further supporting information and discussion on two further behaviors and how these relate and could be realized in neuroWalknet.

Standing

In the Introduction (sect. 2), we mention six motivation units, representing states Stand-Walk, Forward-Backward, Stance-Swing, but did not address examples concerning state Stand. As, however, Standing embraces an important aspect of behavior, in the following, we will briefly sketch cases concerning state Stand that form a necessary complement to walking behavior.

At first sight, standing seems to be a simple subcase of walking, as in the current version of neuroWalknet, just switching the global velocity value to zero is sufficient. However, the following will illustrate the complexity of this behavior, and will show that the structure of neuroWalknet based on motivation units, here Stand and Walk, may provide a relatively simple way to integrate both aspects, standing and walking. Four behaviors concerning the way how to deal with external forces will be addressed.

Biological Experiments: When the intact animal is standing freely and with all six legs each on a 3D force transducer platform, spontaneous changes of the torque in one joint can be observed in all legs, and other torques may change too, but in such a way that nonetheless the position of neither legs would change. This behavior led to the interpretation that in state Stand all 18 joints are controlled by negative feedback controllers each equipped with a position integrator [1, 2]. As a consequence, the body and the leg positions stay absolutely fixed although the torques of the individual legs may change considerably and apparently seem to approaching a minimum value. The network studied by Schmitz and Stein [3] may provide a way to optimize these force distributions between the legs via minimizing cuticular stress.

However, over very long temporal periods another behavioral property, called “flexibilitas cerea”, studied in detail by [4] shows that leg positions may change, too, but very slowly, possibly to minimize the sum of all torques even further (review [5]). When, however, not only—as in the cases mentioned above—the own body weight has to be supported, but stronger, permanent external forces have to be counteracted, further mechanisms are activated. To study this behavior in more detail, the reactions of individual leg joints to disturbances have been recorded, while the body was fixed to a holder. In this situation, first the joint develops a significant torque to counteract the external disturbance, but over time this force is decreasing. This behavior can be interpreted as a negative feedback controller with phasic-tonic dynamics ([4, 6, 7], Simulations: [8]).

As a further case—as in the interim situation between dealing with small loads (in comparison with the own body weight, [1, 2]) and stronger external forces as above [4]—, experiments have been performed, where the external forces where characterized by variable elasticity. When the external elasticity was low (i. e., stiffness high)—similar to case (2)—the joint controller showed a phasic behavior. However, with lower stiffness, the joint kept its position in place for a long time (for details see [9, 10]).

Some of these behaviors have been simulated individually, but not jet integrated into a global structure as neuroWalknet. These could be integrated into neuroWalknet via unit Stand and may thereby in future work combine walking with standing.

Searching

Searching movements have been studied in stick insects with fixed body and partly restrained legs (e.g. [11–13] in detail, but also in free walking animals [14–16]). In neuroWalknet, searching movements are assumed to be controlled by unit Walk and, as there is no ground contact, by unit Swing. Several hypotheses have been discussed concerning the swing movement as such, either as a property of the swing controller with negative feedback that may overshoot the target position if no ground contact has been found followed by a back swing [15], or an intrinsic central oscillatory system implemented in the swing controller [17]. Another model [18] showed that simulation of active reaction could also produce searching-like oscillations. Berg et al. [19] could show that the non-spiking interneuron I4 seems to control search movement (for a discussion, see [20]). As, however, details are not known yet, we did not implement a specific version in neuroWalknet.

References

1. Lévy J, Cruse H (2008) Controlling a system with redundant degrees of freedom. I. Torque distribution in still standing stick insects. J Comp Physiol A Neuroethol Sens Neural Behav Physiol 194:719--733

2. Lévy J, Cruse H (2008) Controlling a system with redundant degrees of freedom. II. Solution of the force distribution problem without a body model. J Comp Physiol A Neuroethol Sens Neural Behav Physiol 194:735--750

3. Schmitz J, Stein W (2000) Convergence of load and movement information onto leg motoneurons in insects. J Neurobiol 42:424–436

4. Bässler U (1983) Neural basis of elementary behavior in stick insects. Springer, Berlin, Heidelberg, New York

5. Pflüger H-J, Büschges A, Bässler U (2021) Historical Review on Thanatosis with Special Reference to the Work of Fritz Steiniger. In: Sakai M (ed) Death-Feigning Insects Mech. Funct. Tonic Immobility. Springer, Singapore, pp 15–21

6. Bässler U (1976) Reversal of a reflex to a single motoneuron in the stick insect Carausius morosus. Biol Cybern 24:47–49

7. Bässler U (1988) Functional Principles of Pattern Generation for Walking Movements of Stick Insect Forelegs: The Role of the Femoral Chordotonal Organ Afferences. J Exp Biol 136:125–147

8. Sauer AE, Driesang RB, Büschges A, Bässler U, Borst A (1996) Distributed processing on the basis of parallel and antagonistic pathways simulation of the femur-tibia control system in the stick insect. J Comput Neurosci 3:179–198

9. Cruse H, Kühn S, Park S, Schmitz J (2004) Adaptive control for insect leg position: controller properties depend on substrate compliance. J. Comp. Physiol. A 190:

10. Schneider A, Cruse H, Schmitz J (2006) Decentralized Control of Elastic Limbs in Closed Kinematic Chains. Int J Robot Res 25:913--930

11. Bässler U (1993) The walking-(and searching-) pattern generator of stick insects, a modular system composed of reflex chains and endogenous oscillators. Biol Cybern 69:305–317

12. Bässler U, Rohrbacher J, Karg G, Breutel G (1991) Interruption of searching movements of partly restrained front legs of stick insects, a model situation for the start of a stance phase? Biol Cybern 65:507–514

13. Nothof U, Bässler U (1990) The network producing the “active reaction” of stick insects is a functional element of different pattern generating systems. Biol Cybern 62:453–462

14. Bläsing B, Cruse H (2004) Stick insect locomotion in a complex environment: climbing over large gaps. J Exp Biol 207:1273–1286

15. Dürr V (2001) Stereotypic leg searching movements in the stick insect: kinematic analysis, behavioural context and simulation. J Exp Biol 204:1589–1604

16. Ebeling W, Dürr V (2006) Perturbation of leg protraction causes context-dependent modulation of inter-leg coordination, but not of avoidance reflexes. J Exp Biol 209:2199–2214

17. Bläsing B (2006) Crossing Large Gaps: A Simulation Study of Stick Insect Behavior. Adapt Behav 14:265–285

18. Bässler U, Koch UT (1989) Modelling of the active reaction of stick insects by a network of neuromimes. Biol Cybern 62:141–150

19. Berg E, Hooper SL, Schmidt J, Büschges A (2015) A Leg-Local Neural Mechanism Mediates the Decision to Search in Stick Insects. Curr Biol. https://doi.org/10.1016/j.cub.2015.06.017

20. Schilling M, Cruse H (2020) Decentralized control of insect walking: A simple neural network explains a wide range of behavioral and neurophysiological results. PLOS Comput Biol 16:e1007804
