# Supplementary material for: neuroWalknet, a controller for hexapod walking allowing for context dependent behavior
Source: PLoS Comput Biol. 2023 Jan 24;19(1):e1010136. doi: 10.1371/journal.pcbi.1010136 (PMC9897571; doi:10.1371/journal.pcbi.1010136)
Supplement: Attachment — Submitted filename: 20122_10_09_ReviewerResponses.docx [file pcbi.1010136.s002.docx]

RESPONSE TO COMMENTS – ALL REVIEWERS

First, we want to thank all the reviewers and the editors for the consideration of our manuscript as we are aware that the paper is quite long. We are in particular happy as the reviewer all share a quite favorable view on our article.

Further, we want to thank you for all the suggestions – we think that many of these helped us to improve the article further. As the two main points raised, we worked through the whole document and tried to clarify the experiments and results. In particular, we elaborated on what is novel in this article and how we realized the quite specific experimental settings used in the biological experiments (this added considerably to the method section, but after reading the reviews we agree with the reviewer(s) that it is important to be more specific on the experimental settings as we have to be very careful to not change our model and adapt the model to a specific experiment). Secondly, we considered how we presented CPGs and contrasted this from our approach as this was raised by multiple reviewers. From our point of view, the first reviewer proposed a nice way to handle this which, actually, didn’t require too much of change in the discussion, but putting this discussion into a better scope and emphasizing how inhibitory connections might have evolved as an early adaptation (for avoiding co-contraction) and later-on more refined CPG-like structures appeared.

We provide a difference document (as a PDF in order to keep the line numbers organized; line numbers mentioned in the following are with respect to this difference document) that highlights the changes made.

<https://www.dropbox.com/s/oep28mceoixav9d/2022_10_13_diff_document.pdf?dl=0>

In the following, we will address the reviewers’ comments one by one.

REVIEWER 1 RESPONSE:

Reviewer #1: This is interesting work but the paper needs substantial revision for clarity.

The core aim of the study is to show that a decentralized neural network for hexapod walking, which has previously been shown to effectively reproduce a number of characteristics of walking behavior in insects (specifically stick insects) can also be shown to reproduce some widely studied phenomena observed in single leg reflexes. This is significant because these phenomena have in the past been taken to be indicative of particular neural control mechanisms but might in fact be just emergent properties under rather specific experimental conditions. The work presented also allows predictions of some new effects that could be observed. Finally, testing the model under these conditions, which it was not explicitly developed to explain, should provide good evidence that it may indeed be a correct account of the mechanism in the insect.

However, the final point to some extent depends on how clear it is that the ‘minor (technical) expansion’ (line 127) to the previous model that has been made in this paper is not in fact careful tweaking to the model designed to produce the desired outcomes under these new conditions. E.g., the fact that the model required some additions to allow the new experiments is not even mentioned in the abstract.

Response: The reviewer raises a crucial issue for this article and we agree with his assessment. It is important to point out that we did not tweak the model into the quite differing experimental situations. Instead, we introduced some extensions (e.g., for additional contexts) that are present in all described simulations. And, further, we carefully setup experimental situations in the simulation. This is now detailed in much more detail in the second section (starting line 169 in the diff document) which is now much more extensive and should clarify this.

Also, several of the experiments being reproduced seem quite complex in terms of the controls applied to the animal (isolated legs, removed sensing, no ground contact, etc.) and it is frequently hard to follow exactly how those conditions have been applied to the model, or how much scope there is within the model to vary the exact implementation until the desired qualitative effect emerges. I think the authors can probably defend their approach against such criticism, but the paper needs to be better presented to do so.

Response: As mentioned above, we now provide much more detail on how we reproduced the settings from the biological experiments in the completely reworked second section.

A final general comment is that I felt the writing could be significantly improved. In particular there seems to be a lot of repetition, with the same point being made several times in subsequent paragraphs. This is often confusing as the reader is not sure if it is the same point or a new (related) point that needs to be understood before proceeding. I have endeavoured to note some specific examples of this problem below. The text also seems to fall between the gap of not explaining enough about the model to be comprehensible without reference to the previous paper, yet explaining too much to be read as a straightforward extension of the work in the previous paper, as it seems to repeat a number of points already made in that paper. Most specifically, the demonstration that ‘CPG-like’ oscillation can occur in the decentralized network under conditions taken to mimic the application of pilocarpine is shown inFig 8 in Schilling & Cruse, 2020, but the current paper is not always clear as to whether the observation of emergent oscillations is a new result here or not.

Response: We tried to improve the writing and went carefully through the text. In particular, we tried to shorten and remove repetitive parts when possible. Secondly, we tried to be more precise when possible.

For the oscillation part: We clarified this through adding early on (line 23, in the Abstract) that this has been shown in the original study and changed the text in the respective section(s).

Specific comments

Comment: We want to thank you for the detailed effort (and connecting to your previous points) we tried to incorporate these suggestions as good as possible. We found them extremely helpful.

Line 22, “Furthermore, such reflexes have been studied while the ganglion was treated with pilocarpine” - as far as I could tell from this paper, the pilocarpine experiments differ from the reflex experiments, they are not a variation of them.

Response: We changed this (see above, now in line 23) in the abstract as well as in the brief description of experiments in the introduction (following line 136).

Line 29 “When experimenting with pilocarpine” - this is an example where the paper sets up the expectation in the reader that the appearance of oscillations is a novel result; and does not refer to what I take to be actually new in this paper, i.e., showing entrainment of these oscillations to sensory stimuli.

Response: see above – has been clarified by adding line 23.

Line 67, it seems odd that the ‘answer’ to the ‘first question’ “how could possible functional elements [of a control system] be defined” is to say, in effect, ‘we will just use some elements we defined earlier’. I find the whole way this part is introduced somewhat awkward, and would prefer they went straight to the point (line 71) that the purpose of this article is to test an existing model against some different behavioural findings.

Response: We reformulated this whole passage and shortened this part of the introduction (line 61 to 73), trying to directly coming to the point.

Line 94, line 98, line 108 example of unnecessary repetition - “Specifically, the focus will be on context dependency” “will in particular focus on context dependency” “In all these experiments the response depends on the context”

Response: We removed repetitions.

Line 97, I think it would be helpful to the reader to explain clearly each of the reflexes to be studied at this point in the paper, rather than describe one and hint at the others. In particular it seems important for motivating the work to explain that the ‘active reaction’ effect has been considered to require explicit mechanistic control, and hence it is very relevant to understand if it might just emerge from the current model. If possible, some kind of illustrations to accompany table 1 would help a reader less familiar with the system to grasp the key points for each experiment. Also explaining the range experiments here, and then, in the relevant results section only briefly recapitulating the specific experiment being reproduced in simulation would make it much easier to follow the results.

Response: We followed the reviewers advise and mentioned all experiments briefly (line 127-144).

Line 111 “behaviour of a (more or less) intact animal has been compared with animals experiencing the same treatment but, in addition...” - what treatment are these (more or less) intact animals experiencing?

Response: This is now answered briefly here in the introduction (line 137) and in detail in the second section (for the pilocarpine treatment see line 425-436).

Line 132 “Furthermore...” these two sentences seem unnecessary, the point has already been made on lines 120-122. Or remove lines 120-122.

Response: Done.

Line 145 “While most control approaches employ one holistic control unit that controls all available degrees of freedom”. I am struggling to think of any control approach in either robotics or biological modelling that fits this description. It seems unnecessary to make this statement.

Response: We have removed this statement.

Line 152 on, it would help if this paragraph was written much more clearly as an explanation of what is common to Walknet and the current model, and what is different. E.g. is the line “Different actions are represented by so called Motivation Units” true of Walknet? As presumably it is not true in Walknet that these are “simple artifical neurons etc.”. I do not understand the sentence “In neuroWalknet, as the main difference, the control circuits as such have been included into these neural circuits, one for each joint.” Do you mean the controllers “have been implemented as neural circuits”; and is the property of “one for each joint” specific to neuroWalknet or inherited from Walknet?

Response: We followed the reviewer’s advise and rewrote this section (lines 181-271): First, pointing out the main principles of Walknet (across many different versions) and then mentioning changes and adaptations in neuroWalknet and in the current version.

Line 169 on, I ended up thoroughly confused about what was new to the model in this paper. Is it including pairs of states (stand/walk, stance/swing, forward/backward) that were in Walknet (line 174) but were not in the previous neuroWalknet (line 180)?

Response: See above – we think your suggestion for reorganization and rewrite should have helped clarifying this.

Line 196 on, is the point being made here that the current simulations are just going to look at the activation of the motor units, and not the actual leg motions produced? I actually found it quite hard to grasp from the paper whether the simulations had been done with full coupling to the simulated body of the stick insect (it appears this was the case from line 817 in the methods?) or how important this was to the results, given these are reported in terms of motor neuron activity only.

Response: We tried to clarify this early-on and make this explicit (starting line 357): *This is directly replicated in our simulation experiments. … Further, as in the biological experiments, in the simulation motor units can be recorded, but cannot activate motor output (“muscles”) and cannot provide feedback to the sensors*

Line 219 on briefly describes the new specific sensory inputs introduced into the model, referring to the methods for details. However, it is not well explained here or in the methods (lines 833 on) how it was decided that the new CS inputs should connect to the Swing/Stance units or the fCO be connected to Stance or to the specific beta joint. Biological data may be lacking but is there a functional justification, beyond that of the specific results that this model is meant to explain?

Response: We added a section in the overview part on the sensory inputs (and how these are simulated), see line 291, following. Further details are given in the experiments in the subsections of the third section.

Results

General comments: I assume the grey/white background to the figures represents swing/stance but this was not stated anywhere I could notice. It is potentially confusing to the reader why the various motor outputs start in such different states before the stimulus is applied. It would also help, for these figures, to include alpha/beta/gamma labels, as in the circuit diagram, for clarity.

Response: We added this information to the figures.

The captions unnecessarily describe details (what the line colours, and graphs in different positions show) which are labelled within the figures; while there is not enough in the captions to highlight the key observations made. Throughout, I felt it might have helped to have an accompanying illustration of the experimental procedure that is being replicated, e.g. showing the animal is intact or missing legs or sensory feedback, where the stimulus is applied, etc.

Response: We shortened the captions and tried to make them more onto the point.

I did not understand why the results for passive state, which are frequently presented first in the sections describing the biological phenomena were not included in the main text of the paper.

Response: This point is completely justified. The idea is to demonstrate the position of unit Stand within the motivation unit network. Therefore, in the first version, we believed that it might be sensible to illustrate the situation by mentioning more details. However, when focusing on the biological data used here, detailed properties of passive state cannot be shown. Therefore, we now moved biological experiments concerning the passive state to Supplement and only briefly addressed motivation unit Stand in the paper to illustrate the general structure.

Fig 2, text from 281, it is not fully clear how the state is set up in the model to be a close reflection of the experiments, but it seems the result is a straightforward consequence of having set up the CS input to trigger transition to stance, plus the opposite direction of motion in stance in backward walking. The most conspicuous change is the decrease in Pro-output (Ret) in forward (backward) walking but this is not mentioned in the text.

Response: This aspect has now been explained in more detail already in Sect. 2, **Sensory inputs** (lines 310 – 317) and **Difference between legs** (from line 367 on), whereas Sect. 3.1 has been shortened.

Line 288 “in both cases, relaxation of CS stimulation leads to activation of the antagonistic muscle” - is this in the simulation or the animal or both?

Response: This sentence referred to simulation but has now been cut.

Fig 3 and related text, why not use the middle leg in the simulation, given that this seems to be the crucial leg in the experiments? It is not very helpful here to label the blue line as FeTi flexion when everywhere in the text this is referred to as the gamma joint.

Response: This is with respect to the original experiments that considered front legs. Now mentioned in the text (line 664-672).

Fig 5 appears to be the same data as fig 4, only to demonstrate matching of a different biological observation. Is there any reason to keep these separate, rather than just to show in fig 4 that there is a match to existing data on the beta joint as well?

Response: We integrated these figures now into a single figure (Fig. 4).

In the interests of a timely return to this review, I will just note that the discussion again contains substantial repetition and could be much more concise.

Response: We went through the discussion and tried to shorten it. In particular for the Active Reation part we rephrased this and integrated this into one section.

One final specific conceptual comment: I am not sure it is necessary to maintain such a strict dichotomy regarding existence/non-existence of a CPG. Indeed, this study and the previous paper support the idea an evolutionary process: first the animal develops a mutually inhibitory network to prevent opponent muscle co-contraction; then it has the possibility to co-opt the implicit oscillatory properties of this network to improve control.

Response: We followed this advise and rephrased it following this reviewer’s advise mostly. We like how he puts this into perspective. This doesn’t require too much change of our comments in general, but phrase these much more carefully and providing a much more open interpretation how these two types of systems could be related (on an evolutionary scale, see lines 1075-1113).

We want to thank the reviewer again for all the detailed comments and corrections. We appreciate very much the time and effort in this careful reading of our manuscript.

REVIEWER 2 RESPONSE

Reviewer #2: This excellent and ground breaking paper examines the potential mechanisms underlying pattern generation and motor control in stick insect walking, focusing upon the context dependence of sensory feedback. The studies use the artificial nervous system, neuroWalknet, including modifications such as local pre-motor interneurons with reciprocal inhibitory connections (in premotor and 'motivational' neuron nets). In a tour-de-force series of tests, the model is used to examine the results of a number of previous biological experiments. Those studies include: the classic reflex reversal and joint specific effects elicited by stimulation of the femoral chordotonal organ; context dependence of sensory feedback in curved walking; and, remarkably, the effects of sensory feedback from sensory receptors indicating force and those signaling kinematic variables on rhythmic activities artificially induced by pilocarpine. In each case, the model is able to reproduce the major findings of the studies and also provide insight into the underlying mechanisms that could be utilized and tested in future biological experiments. Although the details of the reasoning behind each simulation can be discussed and 'picked apart' by others in review, I believe the model results, as whole, are remarkably accurate in reproducing the biological data and generate new testable hypotheses.

This is one of the most important papers to appear in the field of pattern generation and motor control in recent years and offers a different perspective than other studies. While the authors extensively discuss the issues of the existence of CPGs, which is conceptual and open to diverse interpretations, I strongly believe that the paper should be published as it offers new approaches that can be tested experimentally. This is a major advance in the field.

Response: Thank you very much for these kind words and this generous assessment of our work.

I have suggested a number of revisions and corrections to the text that I feel will strengthen the manuscript but use of the suggested text (below) is at the discretion of the authors and editor.

Major (significant) revisions

1. Changing the emphasis of discussion of CPG's - The descriptions of the simulations reported in this study are excellent. However, the discussion of these results focuses upon how the proposed model does not contain elements that are intrinsically rhythmic, i.e. the stimulation does not rely upon a CPG. While that reasoning is valid, I believe that the issue of whether interneurons in the CNS can be conceptually described as a CPG is secondary to the major strength of the paper, i.e. formulating an artificial nervous system that can accurately reproduce a broad range of biological data. I suggest that including elements of the following in the discussion:

Response: We rephrased this part. The first reviewer pointed this out as well and provided a nice alternative to our text. We followed his advise *(“this study and the previous paper support the idea an evolutionary process: first the animal develops a mutually inhibitory network to prevent opponent muscle co-contraction; then it has the possibility to co-opt the implicit oscillatory properties of this network to improve control.”)* and rephrased this part of the discussion which leads to a much more careful phrasing. The new section provides, in our opinion, a much more open interpretation how these two types of systems could be related (see lines 1074-1113). Further, we adapted the respective parts in the introduction and results section in order to put this better into perspective.

2- Critical analysis and modifying discussion of caudal bending - line 208 and elsewhere - It is important that the authors include the following caveat in discussion of experiments using caudal bending of the femur. Although it has been used experimentally in a number of biological experiments, the natural circumstances in which caudal bending would occur are unclear. CS respond to resisted contractions of muscles and caudal bending (simultaneously activating trochanteral and femoral groups of CS) would only result from a very large resisted contraction of the protractor muscle (i.e., encountering an obstacle during the swing phase.) It has been argued that caudal bending occurs transiently at the onset of stance after the tarsus makes contact with the substrate. The only evidence to support that hypothesis is ground reaction forces that are much smaller and shorter in duration than the forces applied experimentally. In all, the findings of massive and potent effects of caudal bending applied to the femur are likely artifactual and do not occur in nature. Although the present study does not directly address these issues, it is important that the authors critically discuss the experiments used in simulations.

Response: We added much more detail in the second section on the sensory inputs (including caudal bending, lines 305-317, as well in line 363-405) and in the experimental third section.

Minor corrections

Abstract - line 14 - better as: This controller allows for walking patterns at different velocities in both forward and backward directions.

line 20 - better as: The reflexes are elicited by stimulation of the femoral chordotonal organ (fCO) or groups of campaniform sensilla (CS).

Response: Both done.

line 29 - The current statement is in a sense misleading and I believe that the authors intend: In simulations of the effects of pilocarpine.

Response: We rewrote the sentence in order to clarify this.

line 75 - better as: Indeed, it allows for control of the basic different aspects of forward and backward walking,

line 88 - clearer as: this would further support the validity of the model.

Response: We removed these sentences (part of a shortening and rewrite of the introduction).

line 144 - delete words: the case of

Response: Done.

line 149 - delete words: still it is shown that

Response: Sentence removed (see above, part of rewrite of introduction).

line 155 - Change 'a leg' to 'that leg'.

Response: Done

line 631 - better as - we developed neuroWalknet, a simulator that is able emulate diverse behaviors

Response: Done.

We want to thank the reviewer for his comments and corrections. We appreciate very much the time and effort.

REVIEWER 3 RESPONSE:

Reviewer #3: The authors actual work represents the further evolution of the Walknet architecture, born on the basis of observations and experiments on the stick insect and now a paradigm for the decentralised control of locomotion, apparently in contrast with the centralised locomotion control, namely CPG. The set of basic rules governing interleg motions, strictly depending on local feedback, later evolved in neuroWalknet in 2020, which took into account other behavioral and physiological results, while negotiating curves or changing locomotion pattern and velocity, stopping and so on.

In this manuscript the attention is focalised on interjoint (intraleg) relations, relying on specific experiments based on stimulation of specific interjoint sensors, like femoral Chordotonal organ or campaniform sensilla. It is shown that the simulated neuroWalknet can reproduce the corresponding experimental results, and generalise them to other legs or joint motions, not yet detected experimentally, working as a simulation/prediction setup to be tested in future experiments on insects.

Moreover, neuroWalknet, at the level of intraleg joint evolutions, can show also context-dependent behaviors.

The paper is nicely written and experiments, as well as simulations, are sometimes so detailed that it is somewhat difficult to follow, at a first reading.

Some main points need to be fixed in order to further refine the paper:

The first one regards the concept of active reaction vs. resistance reflex. As outlined by the authors, resistance reflex, (i.e. negative feedback) or active reaction depend on the insect state (active or passive), and are context-dependent. The authors should specify if the active reaction may also depend on the coupling between neuroWalknet and the dynamically simulated body. This is unclear, since the authors declare that all the simulations were done in an open loop. Is this related only to the stimulation of the specific sensors (femoral Chordotonal organ or campaniform sensilla) or all the simulations are performed without a “body”?

Response: We tried to clarify this: First, we reorganized and mostly rewrote the second section which addresses now in detail how the sensory setup is realized in the simulation. This is, secondly, further detailed in the experimental sections (third section). And, last, we rewrote the discussion on the Active Reaction to clarify this.

The second point is that, since the network was built from a decentralised, feedback-dependent point of view, the authors conclude that there is no need for a central controller for locomotion, as instead widely accepted. Indeed neuroWalknet structure contains basic modules, the “bistable monopoles” organised to control the motor neuron activity, with the ability to generate oscillations in presence of particular stimulations.

If from the one side, CPG, that was born essentially as an open loop control scheme (see Pearson’s or Grillner’s works) a form of sensory feedback was needed to include to account for necessary adjustments coming from the environment.

On the other side, the concept of pure decentralization should be relaxed towards a more flexible architecture, allowing for the possibility to host endogenous oscillations (revealing the emergence of CPGs) which drive locomotion, for example when escape reactions have to be implemented. The neuroWalknet indeed contains such basic modules and the authors should further outline that this scheme would complete, (instead of compete with) the CPG scheme, which, under this view, would represent a specific solution of a wider locomotion scheme whose major assumption is the need for embodiment. Even if there is no learning within this structure, the CPG units would represent learned (fast) solution, to be implemented in specific conditions, within a wider architecture, like neuroWalknet. This issue could be discussed by the authors in the discussion Section.

Response: We think the reviewer raises a very good point. This concurs with the first reviewer and we followed the phrasing of this first reviewer. As a change, we reformulated this in the discussion and tried to be more cautious in discussing CPGs, and, in addition, to point out how these approaches complement each other.

Minor issues:

Fig.1: this figure represents the section devoted to the control of the joints of a single leg. However in the lower central-right hand side of the figure, the green nodes and the boxes are reported and nont mentioned. Please try to at least sketch the role and refer to the literature.

Response: Now added to the explanation (lines 186-199 in the diff document).

Since attention is posed to the control of intraleg joints, a scheme of the leg, reporting the joint and the associated motion, as several times reported by the same authors in other papers, should be inserted into Fig.1. This would allow readers to follow the paper more fluently.

Response: We added a subfigure (1 b)) showing the details of a leg.

Lines 220 etc. please specify why the input “load” seems to have a stepwise form, whereas the fCO is graded, ramp-like. Is this an assumption for the simulation or it is suggested from experiments?

Response: We rephrased this (line 301-304). Furthermore, this is part of a general rewrite and reorganization of the second section in a way that we provide much more detail on how to setup the experiments in simulation.

Line 282 “as soon as the leg is bend”  bent

Response: Done.

The effect of pilocarpine on the PMN module would be better explained, possibly with a figure. In line 229 the authors report that the structure is a soft-WTA, but that a simultaneous input would onset oscillations.

Response: We slightly rephrased this (now in line 425 following) and added a figure showing this detail (new Fig. 5, see as well caption).

We want to thank the reviewer again for all the detailed comments and corrections. We appreciate very much the time and effort in this careful reading of our manuscript.
